# The Role of Indoor Plants in air Purification and Human Health in the Context of COVID-19 Pandemic: A Proposal for a Novel Line of Inquiry

**DOI:** 10.3389/fmolb.2021.709395

**Published:** 2021-06-30

**Authors:** Rania El-Tanbouly, Ziad Hassan, Sarah El-Messeiry

**Affiliations:** ^1^ Department of Floriculture, Ornamental Horticulture and Landscape Design, Faculty of Agriculture, Alexandria University, Alexandria, Egypt; ^2^ Department of Genetics, Faculty of Agriculture, Alexandria University, Alexandria, Egypt

**Keywords:** COVID-19, SARS-CoV-2, indoor plants, air-purification, phytoremediation, humidity, human health

## Abstract

The last two decades have seen the discovery of novel retroviruses that have resulted in severe negative consequences for human health. In late 2019, severe acute respiratory syndrome coronavirus 2 (SARS-CoV-2) emerged with a high transmission rate and severe effects on human health, with 5% infected persons requiring hospitalisation and 3.81 million deaths to date globally. Aerosol particles containing virions are considered the main source of SARS CoV-2 transmission in this pandemic, with increased infection rates in confined spaces. Consequently, public and private institutions had to institute mitigation measures including the use of facial masks and social distancing to limit the spread of the virus. Moreover, the role of air purification and bio-decontamination is understood as being essential to mitigate viral spread. Various techniques can be applied to bio-decontaminate the air such as the use of filtration and radiation; however, these methods are expensive and not feasible for home use. Another method of air purification is where indoor plants can purify the air by the removal of air pollutants and habituated airborne microbes. The use of indoor plants could prove to be a cost-efficient way of indoor air-purification that could be adapted for a variety of environments with no need for special requirements and can also add an aesthetic value that can have an indirect impact on human health. In this review, we discuss the emergence of the COVID-19 pandemic and the currently used air purification methods, and we propose the use of indoor plants as a new possible eco-friendly tool for indoor air purification and for reducing the spread of COVID-19 in confined places.

## The Emergence of the COVID-19 Pandemic

**FIGURE 1:**
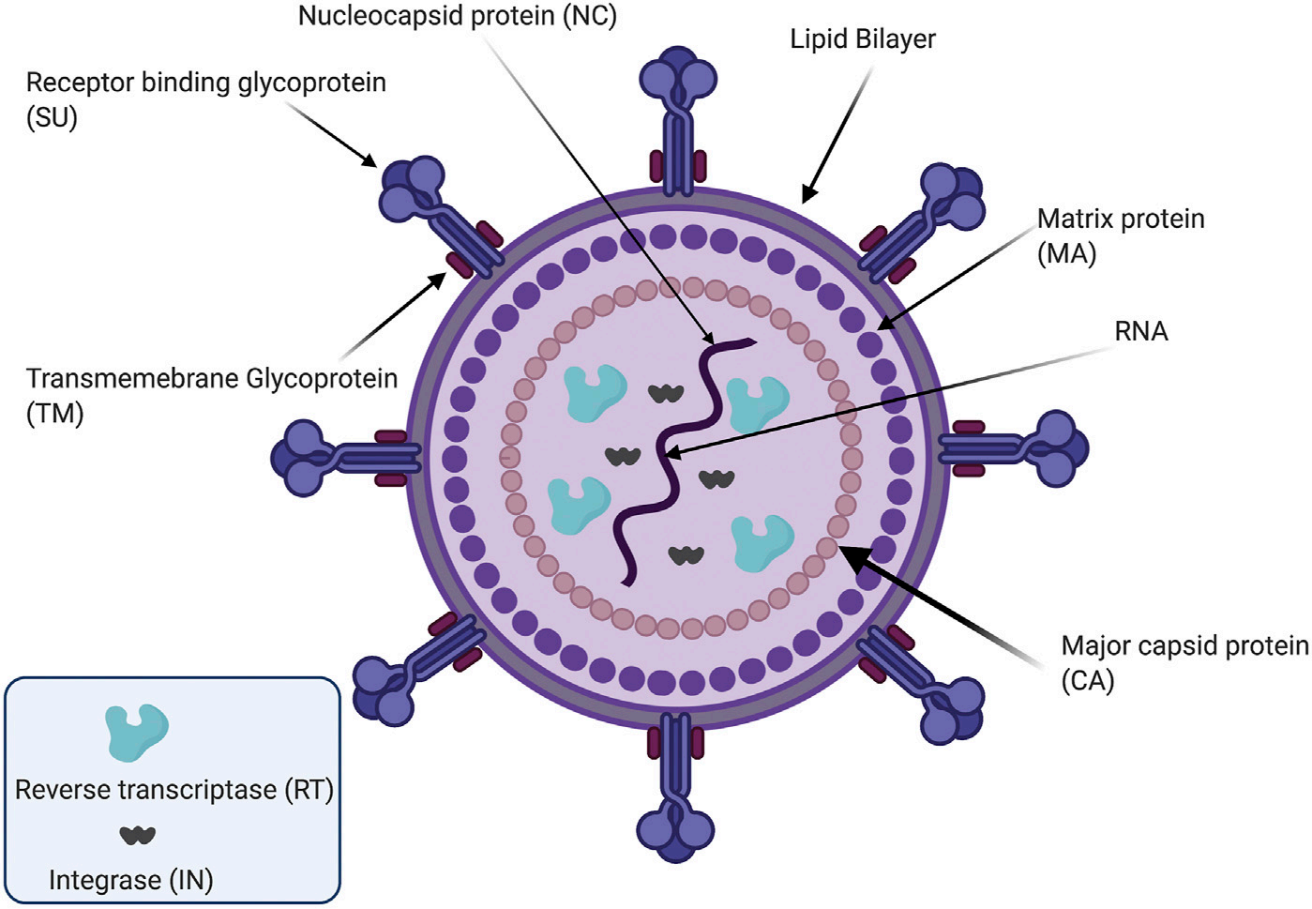
A schematic drawing illustrating the structure of retrovirus. The virus is surrounded by a lipid bilayer membrane. The membrane contains the transmembrane (TM) region and receptor binding glycoprotein (SU). The viral matrix protein (MA) covers the internal side of the viral membrane, while the capsid protein (CA) covers the viral core. The viral core is composed of the viral RNA in addition to the nucleocapsid (NC) protein. The viral core also contains viral replicating enzymes integrase (IN), and reverse transcriptase (RT). This figure is created by BioRender software.

Viruses are small-sized infectious organisms that can be divided into classes according to the type of their genetic material. Retroviruses are a family of viruses in which the ribonucleic acid (RNA) encodes all their genetic information; this family includes severe acute respiratory syndrome coronavirus 2 (SARS-CoV-2), which results in coronavirus disease-2019 (COVID-19) (Petrosillo et al., 2020). Retroviruses possess the ability to invade and infect the host somatic cells and integrate their genetic information into the host genome as a double-strand deoxyribonucleic acid (DNA) (MacLachlan et al., 2017). This process is done using a reverse transcriptase enzyme which can convert their RNA genetic material into DNA inside the host cells via a complementary DNA (cDNA) intermediate (Johnson 2019; Martín-Alonso et al., 2021). The retroviruses family shares a standard structure including an outside envelope with a unique spike protein, which aids in the infection process for each virus, and a single positive strand of RNA that encodes the genetic material including the promoter and regulatory elements essential for the transcription of the viral RNA (Figure 1) (Zhang et al., 2015; Dhama, 2020; Kim et al., 2020; Lu et al., 2020).

Retrovirus family members exhibit a higher mutational rate compared to other viruses of (4.1 ± 1.7) × 10^–3^ per base per cell due to the nature of their genetic information. This mutational activity results in a higher adaptation rate and an increasing number of viruses being discovered every year (Cuevas et al., 2015).

Humankind has been exposed to many pandemic outbreaks that have affected several societies and populations throughout history. However, scientific communities have aided in finding a solution and understanding the source of the pandemic during these harsh times (History 2019). The first pandemic in the 21st century was caused by a member of the coronaviruses family called severe acute respiratory syndrome (SARS), which was discovered in China in 2002 (Cherry and Krogstad 2004). This was preceded in 2012 by an epidemic caused by another member of the same family, the Middle East respiratory syndrome coronavirus (MERS-CoV), first identified in the Kingdom of Saudi Arabia (K.S.A.) after a citizen was admitted to the hospital exhibiting symptoms of severe pneumonia (Fehr et al., 2017). In February 2020, the world was shaken by the WHO announcement of a new pandemic first discovered in China from the same family called SARS-CoV-2 (Brooks et al., 2020; Harapan et al., 2020). This virus was more transmissible than others in the same family as it had a high infection rate of a basic reproductive number R_0_ = 3.1 (Billah et al., 2020; Liu Y., et al., 2020; Read et al., 2020), and a death rate of around 10% (Li et al., 2019). SARS-CoV-2 was shown to be transmitted via horizontal methods including direct contact, droplets, and erosols, or by vertical methods such as surgical procedures and pregnancy (Belser, Rota, and Tumpey 2013; Jiang et al., 2020; Rahman et al., 2020).

A month after the first COVID-19 announcement, the WHO declared the disease outbreak as a global pandemic (Ullah et al., 2020). The SARS-CoV-2 virus has caused threats to public health and resulted in a global travel shutdown all over the world, causing deaths that reached 2,271,180 and confirmed cases 104,370,550 (WHO 6-2–2021) (Liu et al., 2020a). However, current cures and treatments remain insufficiently effective (Liu Q., et al., 2020). Vaccines remain our first line of worldwide virus eradication, and they are limited by storage life and global availability. Hence, governments have applied drastic measures in their countries and required infected persons to be isolated at home, with only severe cases being hospitalized (Kumar and Khodor, 2020; McAleer 2020; Ullah et al., 2020; Vellingiri et al., 2020).

## Overview on the SARS-CoV-2 Virus

The SARS-CoV-2 virus belongs to a monogenic retrovirus family called coronaviridae (CoV) (Sturman and Holmes 1983), whose members can infect up to 200 different hosts (Kasmi et al., 2019). Viruses belonging to this family are spherical, enveloped, and contain a positive-sense, single-stranded RNA (Holmes 1999; Ii et al., 2005; Payne 2017). The coronaviridae family is composed of two subfamilies; torovirinae and coronavirinae; the latter has four genera (Kasmi et al., 2019). SARS-CoV-2 was shown genetically to belong to the genus *Betacoronavirus* along with other coronavirinae respiratory viruses (SARS and MERS). SARS and MERS have infected people worldwide in the last 2 decades with patients exhibiting common cold symptoms resulting in the virus affecting the human respiratory system severely within days of the infection (Li et al., 2019). These two viruses have resulted in the loss of lives in the last 2 decades with a fatality rate of 9.5 and 34.4%, respectively. Studies have shown that SARS-CoV-2 is genetically similar to SARS and MERS, however, the fatality rate of the virus was lower than both viruses with around 2.3%. The reduced severity of the CoV-2 virus contributed to a much higher infection rate (R_0_ = 2–2.5) than the SARS (R_0_ = 1.7–1.9) and the MERS (R_0_ = >1) viruses (Petrosillo et al., 2020), which means that the virus can stay for a long time in the air, increasing the need for air purification as a mitigation method. SARS-CoV-2 has a diameter of (60–15 nm) with a viral genome code for sixteen non-structural proteins (Nsps), in addition to four structural proteins including the 180–200 KDa spike protein (S) which is responsible for the viral subtyping and infection (Gordon et al., 2020; Huang et al., 2020; Kim et al., 2020; Zhu et al., 2020). SARS-CoV-2 has shown genomic and phylogenetic similarity of 75–85% with SARS-CoV (Abdelrahman et al., 2020; Hu et al., 2020; Uddin et al., 2020), especially in the S gene and receptor-binding domain (RBD) (Andersen et al., 2020; Zhou et al., 2020). The glycoprotein spikes on the outer surface of coronaviruses are responsible for the attachment and entry of the virus to host cells (Shereen et al., 2020), as they bind to the Angiotensin-converting enzyme 2 (ACE2) receptor, that is expressed in many body organs such as lungs, kidney, heart and gastrointestinal tract (Hamming et al., 2004; Tikellis and Thomas 2012; Roca-Ho et al., 2017; Davidson et al., 2020; Samavati and Uhal 2020). This is mediated by the binding domain of the protein (Tai et al., 2020); this is then followed by fusion of viral membrane (Walls et al., 2020), which will subsequently release its genetic material in the cytoplasm of the host to be translated (Astuti 2020).

Primarily respiratory disease transmission occurs within 5–6 days of the infection, COVID-19 patients show symptoms that vary from dry cough, fever, breathing difficulties, fatigue, headache, diarrhea, sore throat to severe pneumonia, which increase the need for bio-decontamination (Hosseini et al., 2020). Due to the high mutation rate of the virus, new variants of the SARS-CoV-2 virus are being discovered and named according to the countries in which the virus has been discovered such as (United Kingdom, Brazil, South Africa, and recently in India), complicating viral containment efforts (Callaway 2020; Jia et al., 2020; Korber et al., 2020; Desai et al., 2021; Zhou et al., 2021).

Molecular methods such as the use of real-time polymerase chain reaction (RT-PCR) to detect the virus genome are considered the standard methods in the diagnosis of this disease. Other rapid detection methods are also used to detect the presence/absence of the antibodies produced by the immune system upon infection; this method is considered as the most expensive and time effective. New diagnostic methods are being developed every day to enhance the detection specificity and accuracy and to aid in restricting the virus spread via early and easy diagnosis among with the population (Islam and Iqbal 2020).

## The Role of air Purification in Fighting the COVID-19 Spread

The SARS-CoV-2 virus has been shown to spread via three main routes; erosols, which are relatively small airborne microdroplets (1–4 µm), large respiratory droplets (>5 µm), and close contact with infected surfaces and people. The latter two sources of transmission can be handled via disinfection of surfaces and the use of proper protective equipment. Small erosols droplets, however, are harder to contain with standard sterilization methods and are considered to be the main source of spread, with reports of virion-containing droplets being able to travel in the air and remain viable for around 3 h (Pagliano and Kafil 2020). Most respiratory infections are transmitted via respiratory droplets larger than 5 µm when coughing and sneezing, that are then deposited onto surfaces (Siegel et al., 2019). Pathogens suspended in erosol particles smaller than 5 µm can remain airborne and can deposit in the lower respiratory tract, while those in particles with size ranging from 6–12 µm are deposited in the upper respiratory tract (Brown et al., 1950; Harper and Morton, 1953; Fennelly 2020). Many studies have showed that pathogens and viruses can be found in small particles less than 5 µm (Stelzer-Braid et al., 2016). A recent study was able to detect viral presence in hospital air samples in isolation rooms via RT-PCR, with detected virions in particles with a size range of 1–4 µm and >4 µm (Liu et al., 2020b; Chan et al., 2020; Karia et al., 2020; Rahman et al., 2020). Another study revealed that more than 60% of the air samples from COVID-19 patient rooms contained the virus (Santarpia et al., 2020).

For all the above reasons, critical mitigation measures were put in place to control the spread and control the transmission rate of COVID-19, including closure of high-traffic institutions such as schools and universities, instituting social distancing to decrease exposure to respiratory droplets with COVID-19; use of facial masks to cover nose and mouth, especially when physical distancing cannot be achieved (as in shared transport); reducing the number of workers per square unit; and enhanced ventilation, air filtration, and conditioning to reduce the number of virus-containing droplets and microdroplets in a given environment. Further mitigation measure included advising sick individuals to stay home to avoid potentially infecting others, liberal use of hand-washing and alcohol containing sanitizing gels, and covid-testing where available to identify individuals infected with COVID and potentially infected close contacts (Health et al., 2020; Sandle 2020; Bazant and John, 2021). Additional measures were also needed in institutions to ensure a pathogenic-free environment such as surface cleaning and air purification (Sandle 2021). Many traditional methods were employed to filter the air, additionally, several companies are using artificial intelligence and mechanized systems to bio-decontaminate and clean the area in a time-cost efficient manner (Luengas et al., 2015; Ethington et al., 2018; Garfin 2020; Menut et al., 2020; Muhammad, Long, and Salman 2020; Mukhtar et al., 2020; Megahed and Ghoneim 2021). Researchers also implemented a highly sensitive camera to monitor microdroplets that may transmit viruses providing data that enabled organizations and scientists to guide people on how to protect themselves against disease transmission (Noti et al., 2013).

Practices such as good air ventilation, avoidance of air recirculation, minimization of the number of people in indoor areas and air filtration techniques have been proven to aid in the reduction of the virus spread (Morawska et al., 2020). Filtering the air from virion-containing erosols is especially important in hospitals and COVID treatment units. This can be done by the use of filters such as high-efficiency particle arrestance (HEPA) filters, the highest class of which can trap erosol particles of 0.3 µm diameter with 99.97% efficacy according to (ISO 29463-1:2011, 2017; Nazarenko 2021) (Nazarenko 2021). While effective such units are impractical in most private residences and small-scale commercial shops due to the high cost of units needed to filter air in large open spaces. Ionization devices can also be used to clear the air from virion-containing droplets. Hagbom et al. have shown that ionizing devices clear influenza viruses from the air, trapping the virion-containing particles by creating negative ions that collide with particles of a size range of 35 nm to 10 μm. There results showed that the ionizer device prevented the infection spread with high efficacy in pigs (Hagbom et al., 2015). Also, Suwardi and colleges demonstrated that plant-based ionizers can aid in reduction of erosol-containing virions indoors (Suwardi et al., 2021). However, the wide use and potency of these air ionizing techniques are still not fully investigated. Air bio-decontamination via Ultraviolet (UV) radiation is another method that can be applied to clear the air and surfaces for SARS-CoV-2 virions. UV-C light (200–280 nm) has the ability to damage viral RNA and proteins of the SARS-CoV-2 virus in liquid, solid, and air media (Raeiszadeh and Adeli 2020). The use of this method of air bio-decontamination is limited due to human safety concerns, thereby restricting the use of this method. Also, negative pressure air flow in hospital wards and rooms were shown to reduce the SARS-CoV-2 airborne presence (Pagliano and Kafil 2020). Table 1 summarizes the use of air filtration, UV sterilization and ionization techniques to filter and clear the air of bacteria, fungi, and viruses.

**TABLE 1:** a comparison between the most common methods of air filtration and purification that are used to fight bacteria, fungi and virus spread.

	HEPA filters		UV radiation	Ionization
Mode of action and properties	It functions by the combination of three aspects. First, outer filters that work like sieves to stop the larger particles of dirt		Air disinfection using 254 nm UV-C is an effective tool for inactivating viral erosols. Air microbes, genetic material and protein absorption the UV light resulting in severe cellular damage to the organism Walker and Ko (2007)	It can prevent transmission of airborne viral infections. The ionization device consists of a small portable ionizer, where a sampling cup of positive charge is attached to the ionizer attracting negative particles from the air
	A concertina filter–a mat of very dense fibers–forms the middle layer which traps smaller particles. These are designed to remove 90% of particles from the air. The inner part catch particles as they pass through in the moving air		Air cleaners alone or combined with upper-room air Ultraviolet germicidal irradiation (UVGI) can purify the air by removing bioaerosols at significant rates in high-exposure environments such as hospitals, correction facilities Kujundzic et al. (2006)	Higher numbers of viral particles detected on the active ionizer compared to the inactive ionizer led to the conclusion that this technique can efficiently capture and collect viral particles from the air Hagbom et al. (2015)
	Different grades of HEPA filters differ in their ‘efficiency ratings’. The most used HEPA filter is the H14 filter, which is designed to eliminate 99.997% of particles from the air Sandle (2013)			
	The performance of the HEPA filter system depends on its setting and position. Nevertheless, the use of a mobile HEPA filter system seems a good alternative to use when no ventilation options are available Bluyssen et al. (2021)			
Efficiency	Commercial HEPA filters (99.95%) retain more than 9.996% of actinophage particles Roelants et al. (1968), Dee et al. (2006), Christopherson et al. (2020)	Titanium dioxide (TiO_2_)-coated HEPA filters were shown to be able to remove 60–80% of airborne spore-forming bacteria and fungi Chuaybamroong et al. (2010)	Exposing viral particles to UV-C for 1 s is lethal and possesses the potential to inactivate the viral particles Sabino et al. (2020)	Air ionizers are capable of purifying the air of fine and ultra-fine particles with high efficiency Grabarczyk (2001), Uk Lee et al. (2004), Shiue et al. (2011), Pushpawela et al. (2017)

While the previously mentioned air purification and filtration methods are considered effective, there are some drawbacks that limit their widespread use in homes, schools, supermarkets, and small organisations. Indoor plants can be used as an unconventional method to clear the air from biological and non-biological contaminates as an environmental, and cost- and user-friendly alternative.

## The Role of Plants in air Purification

**FIGURE 2:**
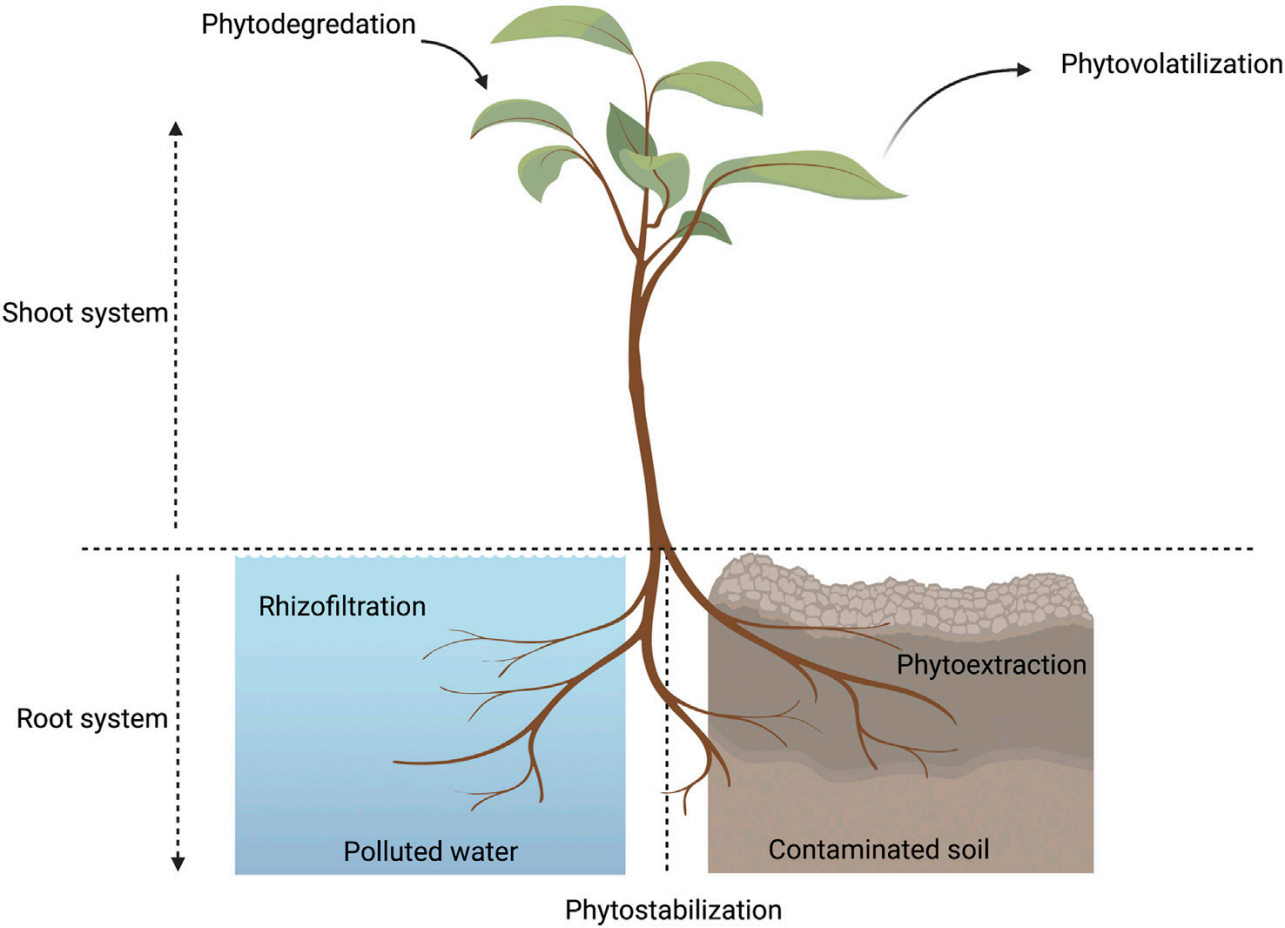
A diagram showing the different phytoremediation techniques used by plants in different environments. Phytoremediation techniques are divided into five main areas. 1) Phyto-stabilization is immobilization of contaminants and pollutants in the soil, consequently reducing their bioavailability and decreasing the risk of leaching into water or spreading in air (Morikawa and Erkin 2003). 2) Phytoextraction is carried out by pollutant accumulation in plants (Lee 2013), 3) Phytodegradationis is the conversion of toxic pollutants into less harmful and non-toxic materials (Cunningham et al., 1995; Newman et al., 1997; Lee 2013), 4) Rhizo-filtration (Phyto-filtration) involves either plant roots (rhizofiltration) or plant shoots (caulofiltration) or seedlings (blastofiltration) eliminating pollutants from water or wastewater (Mesjasz-Przybyłowicz et al., 2004; Yan et al., 2020), 5) Phytovolatilization is the conversion of toxic volatile elements into less toxic forms after being taken up from the soil and consequently released into the air through transpiration process via leaves (Mahar et al., 2016) This figure is created by BioRender software.

Plants are used as an efficient cleaning system for the environment in a process known as “Phytoremediation”, which can be done via various techniques in which plants clear the environment from pollutants (Figure 2) (Pilon-Smits 2005). Indoor plants are considered to be natural air filters as they can purify air through different methods: absorption, dilution, precipitation, and filtration (Kim et al., 2018; Lee, Hadibarata, and Yuniarto 2020). A well-known process carried out by plants is photosynthesis in which plants clean the air through taking in carbon dioxide and releasing oxygen. Respiration is another process where plants absorb oxygen and release carbon dioxide. Through photosynthesis and respiration, the air goes in and out from the stomata, as they are considered the main apparatus that plants use in the absorption and filtration mechanisms (Jones 1998). Plants can absorb airborne molecules and restore the ecological balance in the air (Agarwal et al., 2018). In addition, plants can purify the air from pollutants such as carbon dioxide, volatile organic components (VOC), carbonyl, particulate matter, organic compounds, nitrates, sulfates, ammonia, calcium, ozone, and carbonate. Indoor plants can be considered as a low-cost solution that reduces the levels of indoor pollutants and minimize human exposure to many harmful compounds (Pegas et al., 2012; Soreanu et al., 2013; Abbass et al., 2017; Parseh Iman et al., 2018; Wei et al., 2021).

Indoor relative humidity (RH) is considered an important component which causes an interaction between humans, viruses, and plants. Recommended interior humidity levels for human comfort usually range between 30 and 60% (Fan et al., 2017) and 40–60% shows to prevent viral transmission (Audi et al., 2020). Research has shown that plants are capable of absorbing tiny airborne water vapor particles and have the ability to improve indoor humidity (Jeong et al., 2008; Pérez-Urrestarazu et al., 2016). Concerns were raised, that indoor plants transpire through stomata releasing tiny water droplets to the surrounding air, increasing humidity; however, at high humidity, the transpiration rate by plants decreases maintaining the interior humidity at suitable levels for humans. Pegas and colleagues stated that humidity is highly correlated with the abundance and presence of molds, bacteria, mildew, and other biological pathogens leading to a drastic impact on human health (Pegas et al., 2011). Plants are known for the potential to enhance the humidity through foliar water uptake at high humidity, thus reducing bioaerosols (Lohr and Pearson-Mims 1996; Limm et al., 2009; Gawrońska and Bakera 2014; Brilli et al., 2018; Chiam et al., 2019). Henceforth, plants can control air humidity maintaining it at intermediate levels which lower the viability of SAR-CoV-2 in liquid erosols that decrease the efficiency of viral transmission.

Plants can release in the air small quantities of secondary metabolites and their derivatives, such as polyphenols and alkaloids. This process is considered one of the plant's techniques to interact with the surrounding environment called Allelochemicals (Mushtaq et al., 2019). Those compounds were reported to have antimicrobial activities and can interact with airborne microbes close to the plant (Yang H. et al., 2012; Othman et al., 2019).

Experiments were performed showing the reduction of airborne pathogens by plants, as Wolverton et al. reported that indoor house plants are competent to reduce airborne microbes by 50%, compared to indoor spaces lacking plants. Their data showed that hydroponic planter system containing 15 different houseplant species were able to directly and indirectly suppress the growth of airborne microbes compared to the plant-free room, however they did not mention a specific species and no viruses were tested. The study discussed those volatile substances released by houseplants might be the main reason in controlling the airborne microbe population in plant-included environments (Wolverton and Wolverton 1996). A recent study showed that plants could significantly decrease air microbiomes when compared to plant-free sites, providing a healthier environment and reducing the exposure risk of airborne diseases (Li et al., 2021). Another study was conducted showing evidence that plants can reduce fine particles. In 2016, Stapleton evaluated 11 different household plant species, which showed a significant reduction of ultrafine particles in the indoor environment (Stapleton and Ruiz-Rudolph 2018). Table 2 summarizes different indoor plant species that were proven to reduce airborne pathogens and air pollutants.

**TABLE 2:** Household plants that have been tested to reduce airborne pathogens and pollutants.

Scientific name	Common name	Family	Pathogen	Other indoor pollutants	References
*Ficus benjamina*	Weeping fig	Moraceae	Bacteria, Actinomycetes and Mold	Formaldehyde	Kim et al. (2008), Schmitz et al. (2000), Weidener and Teixeira da Silva (2006), Wolverton and Wolverton (1993), Wolverton and Wolverton (1996)
				Volatile organic compounds	
*Ficus alii*	Alii ficus	Moraceae	Bacteria, Actinomycetes and Mold		Wolverton and Wolverton (1993), Wolverton and Wolverton (1996)
*Spathiphyllum* sp.	Peace lily	Araceae	Bacteria, Actinomycetes and Mold	Benzene	Wolverton and Wolverton (1996), Parseh et al. (2018b)
*Chrysalidocarpus lutescens*	Areca palm	Arecaceae	Bacteria, Actinomycetes and Mold	Ammonia	Wolverton and Wolverton (1996), EL Sayed (2020), Bhargava et al. (2021)
				Formaldehyde
				Total volatile organic compounds (TVOCs), CO_2_, and CO
*Dracaena fragans “*Massangeana”	Corn plant	Asparagaceae	Bacteria, Actinomycetes and Mold	Benzene Ozone	Wolverton and Wolverton (1996), Chauhan et al. (2017), Aydogan and Cerone (2021)
				Toluene Xylene Formaldehyde Trichloro- ethylene
*Rhapis excelsa*	Lady palm	Arecaceae	Bacteria, Actinomycetes and Mold	Formaldehyde	Wolverton and Wolverton (1996), Schmitz et al. (2000), Aydogan and Cerone (2021)
*Dracaena dermensis* “Warneckei”	Warneckei	Asparagaceae	Bacteria, Actinomycetes and Mold	Toluene	Godish and Guindon (1989), Wolverton and Wolverton (1996), Mosaddegh et al. (2014)
				Xylene
				Benzene
				Ethylbenzene
				Formaldehyde
*Deiffenbachia* “Exotica compacta”	Dumb cane	Araceae	Bacteria, Actinomycetes and Mold	Toluene	Wolverton and Wolverton (1996), Sharma et al. (2018)
				Xylene
*Deiffenbachia camille*	Dumb cane	Araceae	Bacteria, Actinomycetes and Mold	Toluene	Wolverton and Wolverton (1996), Sharma et al. (2018)
				Xylene
*Philodendron domesticum*	Philodendron	Araceae	Bacteria, Actinomycetes and Mold	Formaldehyde	Wolverton and Wolverton (1996), Sharma et al. (2018)
*Epipermum aureum*	Golden pothos	Araceae	Bacteria, Actinomycetes and Mold	Benzene	Wolverton and Wolverton (1996), Gong et al. (2019)
*Syngonium podophyllum*	Arrowhead vine	Araceae	Bacteria, Actinomycetes and Mold	CO_2_	Wolverton and Wolverton (1996), Parseh et al. (2018b)
				Volatile organic compounds (VOC)
				Benzene
*Sanseveria frifasciata* “Laurenetii”	Snake plant	Asparagaceae	Bacteria, Actinomycetes and Mold		Wolverton and Wolverton (1996), Seminar (2012), Sharma et al. (2018)
*Codiaeum varigatium*	Croton	Euphorbiaceae	Bacteria, Actinomycetes and Mold	Toluene	Wolverton and Wolverton (1996), Sriprapat et al. (2014)
	Ethylbenzene				
*Cyperus alternifolius*	Umbrella grass	Cyperaceae	Bacteria, Actinomycetes and Mold		Wolverton et al. (1989), Wolverton and Wolverton (1996)

There is lack of experimental evidence that indicated plants having a direct role in the reduction of virus transmission, through controlling the environment. Few approaches were conducted to provide evidence that plants are capable of affecting airborne microorganisms, including bacteria and fungi (Wolverton and Wolverton 1996; Pegas et al., 2011; Li et al., 2021); however, as yet none of these approaches tested the direct impact of indoor plants on virus survival and transmission. Further investigations are still required to elucidate the mechanisms by which different plants can reduce airborne microbes and to specify which plants would be best used to reduce bacteria and viruses from the air to limit indoor disease transmission.

## The Potential Role of Indoor Plant in Fighting COVID-19 via Regulation of Humidity

**FIGURE 3:**
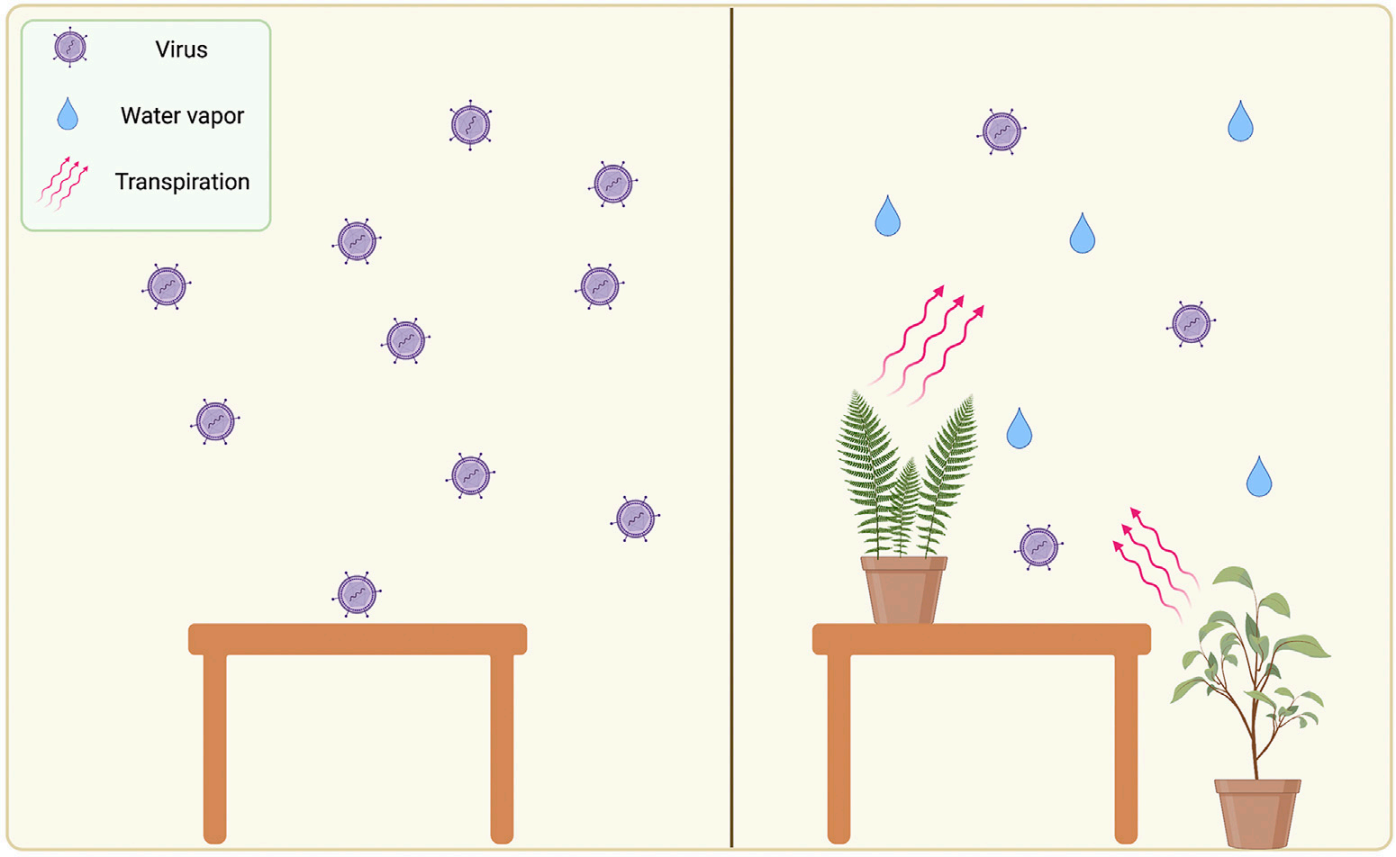
illustration of the potential role of indoor plant in reducing airborne viruses via regulation of humidity. Left section represent a plant-free environment with low relative humidity and high viral transmission. Right section represents effect of plants on increasing the humidity and reducing the viral transmission. This figure is created by BioRender software.

The most probable role that plants can play in reducing SARS-CoV-2 transmission is via modulating humidity in the indoor environment. Studies have shown that in humidity levels ranging from (40–60%) influenza virus transmission decreased in genie pig, concluding that the virus became deactivated in these conditions (Yang Wan et al., 2012; Bhattacharyya et al., 2015). Also, the stability of the virus decreased in these conditions (Audi et al., 2020), via affecting the virus lipid envelope (Tang 2009). Attempted studies evaluated the effect of relative humidity on SARS-CoV-2 transmission, concluding that the virus transmission rates is reduced in high humidity conditions (Wang et al., 2020), also a study found that SARS-CoV is less viable at relative humidity >50% (Chan et al., 2011). Other environmental factors might be involved in viral transmission such as temperature, light, airflow, and other unstated factors. As plants modulate indoor humidity in levels above 30% (Kerschen et al., 2016), we propose that the use of indoor plants can regulate humidity in confined places, which will subsequently decrease the stability of the SARS-CoV-2 in air particles and reducing its transmission rate. The proposed role of indoor plants is described in Figure 3.

## The Role of Indoor Plants on Human Health

Plants play one of the prominent roles that increase the survival of humankind that is living healthy, which causes an enhanced immune system capable of fighting diseases. This role could be attained by eating balanced nutritious food and living in a healthy environment (EEA (European Environment Agency), 2020; Mathus-Vliegen 1995). Plants have been a key element in the human environment that directly and indirectly improve human health (Deng and Deng 2018). Directly, by providing humans with oxygen and food, plant extractions have been widely used in the pharmaceutical industry to aid in disease prevention and treatments (Ribnicky et al., 2002). While indirectly by improving the environment through air-purifying and removal of air pollutants (Qin et al., 2014). Additionally, indoor plants such as lemon balm and hyssop can be used as home remedies to aid in managing the symptoms of cold and flu (Doukani et al., 2021; Judžentienė 2016) Also, plants have a psychological effect that impacts human productivity and destressing that affects human immunity (Bringslimark et al., 2009). Indoor plants have been known to add an esthetic value to their surroundings. However, their effect on the human immune system and health, as well as psychological effects have not been fully investigated and quantified. A study has shown that indoor plants such as rubber tree, English ivy, and spider plants have increased the performance of students in classrooms (Kim et al., 2014). Another study has shown that the presence of indoor scented and unscented plants such as lavender, poinsettia, alocasia rhizome, and apple geranium enhanced human comfort (Qin et al., 2014). Moreover, plant photosynthesis has been shown to improve air quality by producing negative air ions. Additionally, plants remove pollutants, and volatile organic components (some examples are shown in Table 2); plants were also found to regulate temperature and humidity (Deng and Deng 2018). Overall, the presence of indoor plants has many benefits on human psychological health and enhancement of air quality. Nevertheless, the effect of indoor plants on human biological functions is not fully studied due to a lack of quantification methods.

## Future Perspective of the Role of plants in Fighting SARS-CoV-2 Spread

The hypothesis illustrated above shows the need for further investigations and experiments to be conducted to prove the role of plants in reducing viral transmission not only specific to SARS-CoV2 but as a general prospective to improve human health. As mentioned in previous sections, we suggest that indoor plants can serve as an economically and environmentally friendly solution to reduce SARS-CoV-2 spread in confined spaces, which can be used in low-income countries and households as a cheap alternative to complicated filtration systems. This suggestion could be achieved by enchaining the indoor environmental conditions to a much less friendly environment to the virus by increasing the humidity. However, this is a novel idea and studies are needed to confirm our suggested hypothesis. Studies investigating the effect of indoor plants on air quality are limited due to several reasons. For instance, the choice of the experimental site and the experimental setup, as several factors can affect the results; in addition, fine-tuned experimental designs to achieve accuracy incur high costs. These reasons have created a vast gap between theoretical bases and implementation. This lack of information is disappointing as the potentially discovered data might have an enormously beneficial effect on human health by protecting against harmful diseases and limiting viral transmission rates. Scholars and researchers need to establish a standardized protocol for a controlled indoor computerized experiment that mimics the indoor environment and implements artificial intelligence technology to monitor all factors. This protocol can minimize data errors and provide useful research guidelines that will eventually lead to a healthier human environment. In brief, implementing indoor plants to reduce SARS-CoV-2 transmission is an idea worth pursuing in the near future with a well-design experiment and controlled parameters to aid in our fight against the COVID-19 pandemic.

## Conclusion

The COVID-19 pandemic spread has caused drastic changes in policies worldwide; reducing human contact has become preferable, and air bio-decontamination and surface sterilization have become necessary to control the high transmission rate of the virus. Additionally, studies have shown that the virus can stay for a long time and travel in the air in large and small droplets; consequently, high viral contamination rates were found in the air of poorly ventilated areas with recycled air such as hospitals. For all these reasons, air purification and bio-decontamination techniques are recommended to be used in all public and private indoor spaces to contain the spread of the virus in areas with poor ventilation. Especially as the virus could remain in the human population for a longer time than expected due to new strains being discovered worldwide which may escape the immune response. Air purification via filters, UV radiation and ionization could be used to clean the air in large companies and facilities. However, some drawbacks can limit using these techniques, such as high costs and the need for specialized maintenance, making these methods not applicable in rural areas, homes, developing counties, and low budget facilities. Therefore, a novel low-cost air purification method is highly preferable. The answer to this could be the use of the indoor plant to purify the air. Several studies have shown that indoor plants enhance air quality, remove pollutants, and reduce bacterial and fungal infection spread, none of which were on airborne viruses. The techniques by which plants purify the air are not fully understood, and limited research information is available discussing their role in controlling viral transmission. Furthermore, the propose role of plants regulating relative humidity in confined places could be considered an alternative solution that can be used to reduce the viability of SARS-CoV-2 and add esthetic value to the surrounding environment. In conclusion, more research in this area is required in the search for methods of lowering transmission rates in low-budget places, especially as the indoor plants were found to increase human comfort and enhance overall human health.
